# Different facets of compulsive buying among Chinese students

**DOI:** 10.1556/JBA.3.2014.4.5

**Published:** 2014-12-18

**Authors:** SHUANG LI, ALEXANDER UNGER, CHONGZENG BI

**Affiliations:** ^1^School of Psychology, Southwest University, Chongqing, China; ^2^Institute of International Management Studies, University of Applied Sciences, Ludwigshafen, Germany

**Keywords:** compulsive buying, German Compulsive Buying Scale (GCBS), Chinese university students, dimensionality of compulsive buying

## Abstract

*Background and aims:* Compulsive buying is a severe phenomenon, especially among younger consumers. It is well documented in Western industrial societies like the USA and Germany, and nowadays an increasing interest in compulsive buying in non-Western countries is on the rise. *Methods:* In the current study, we measured the prevalence of compulsive buying tendencies among Chinese female and male students by using a Chinese translation of the German Compulsive Buying Scale (Raab, Neuner, Reisch & Scherhorn, 2005). We examined the influence of gender, location and age using ANCOVA, and binary logistic regression. *Results:* Factor analysis identified three factorial dimensions of compulsive buying tendencies which are impairment of impulse control and reactive or compensatory aspects, reduced rationality according to money spending, and post-purchase guilt. Our results indicated that about 6.7% of the sample shows a compulsive buying pattern, and that females are more affected. For location, a geographic difference between Chongqing and Fuzhou was found for the overall compulsive tendencies, but not for the percentages of compulsive buyers. *Conclusions:* In sum, the existing study provides evidence that Chinese consumers have a factorial structure which differs somewhat in compulsive buying from Western samples. Observations about gender and location were considered. These findings give a deeper understanding of China’s compulsive buying behavior.

## INTRODUCTION

To China compulsive buying is quite a new issue of research. Most previous research on compulsive buying dealt with the prevalence, causes, and possibilities of therapy in the maturing Western industrial societies (Workman & Paper, 2010). Under the background of a globalized consumer culture, the interest in research on compulsive buying in non-Western countries, such as threshold countries, new industrial countries like China, or developing countries like Pakistan, has increased over the last decade (Li, Jiang, An, Shen & Jin, 2009; Saleem & Salaria, 2010; Shahjehan, Qureshi, Zeb & Saifullah, 2012).

### Theoretical backgrounds

The American Psychiatric Association classified compulsive buying in the *Diagnostic and Statistical Manual of Mental Disorders* (DSM-5, 2013) as impulse control disorder (ICD) not classified elsewhere. McElroy, Keck, Pope, Smith and Strakowski (1994) defined compulsive buying as a maladaptive preoccupation with buying that is experienced as irresistible and frequently followed by buying items that are not used or needed. These preoccupations impair social functions and may cause financial problems. O’Guinn and Faber (1989), in their theoretical foundation of compulsive buying, complemented the responsive character of compulsive buying to negative events or feelings. Like O’Guinn and Faber, many authors have emphasized the reactive character of compulsive buying (De Graaf, Wann & Naylor, 2005), and some (McElroy, Phillips & Keck, 1994) have criticized the classification as ICD and assumed that compulsive buying should instead be understood as obsessive-compulsive disorder (OCD) because the buying process for compulsive buyers is an attempt to neutralize negative feelings. Supporting observations were reported by Faber and Christenson (1996).

Therefore, we can summarize three main characteristics of compulsive buying from these distinctive definitions: frequent preoccupation with buying, experience of irresistible urges for buying called impaired impulse control, and the attendant adverse consequences.

### How does compulsive buying emerge?

Many studies have dealt with the role that personality factors play in compulsive buying (Mendelson & Mello, 1986; Scherhorn, 1990). Over the last decade, compulsive buyers have been gradually found sharing specific personalities and characteristics, of which low self-esteem and proneness to depression were the most noticeable. Many studies show that compulsive buying is positively correlated with low self-esteem (Black, 2007; Chang & Arkin, 2002; Hanley & Wilhelm, 1992; Yurchisin & Johnson, 2004) and to depression (Black, 2007; Kyrios, Frost & Steketee, 2004; Lejoyeux, Haberman, Solomon & Adès, 1999; Lejoyeux, Tassain, Solomon & Adès, 1997). Furthermore, individuals who show lower levels of self-control (Faber, 2004; Faber & Vohs, 2004) – namely lower levels of impulse-control (Christenson, Faber & de Zwann, 1994), higher levels of anxiety, arousal, and obsessions – also have a higher risk for compulsive buying (Black, 2007; McGoldrick & Pieros, 1998; O’Guinn & Faber, 1989; Scherhorn, Reisch & Raab, 1990; Schmitz, 2005). Besides the theorized and observed personality aspects, other approaches for explaining compulsive buying were put forward such as biological-based explanations, sociocultural theory, social learning theory, and cognitive theory (cf. for an overview Workman & Paper, 2010).

### Compulsive buying in China

Compulsive buying is not a new topic in the Western world. Many researchers, including economists, psychologists, and even biologists, have focused on the prevalence, possible causes, following financial problems, and suitable therapies (Kuzma & Black, 2006). In recent years, an increasing focus on Chinese compulsive buying behaviors has emerged.

One possible reason is the integrated consumer culture brought on by globalization. Although advertising and enhanced materialistic values cannot be understood as the mono-causal factors causing compulsive buying, they do play an important interacting role, along with personal factors such as deficiencies in personality, social relationships, and personal well-being. The increasing per capita GDP of the Chinese population over the last few decades and the availability of a wide range of products also provide a high possibility of compulsive buying behaviors in China. Additionally, electronic payment systems such as credit cards have become another inducement to compulsive buying. Raab (2000) showed that electronic payment systems have indeed facilitated higher spending rates and underestimated consumption rates by the consumers (cf. for the psychological consequences of different payments systems Ariely & Silva, 2002). In a certain sense, these factors offer opportunities to the consumers, especially to young consumers, to learn and establish patterns that help satisfy their need for stimulation and compensate for the aforementioned deficiencies. This, in return, can lead to buying compulsive-ness and overconsumption.

Several authors argue that the etiology of compulsive buying begins in childhood and can be traced back to a distorted development of personality and autonomy (Scherhorn, 1990; Scherhorn et al., 1990). According to Kuzma and Black (2006), the onset of compulsive buying disorder is generally from late adolescence to early adulthood, and this period from late adolescence to early adulthood is also important for the formation of one’s personality (Arnett, 2007). Some studies have further shown that young consumers are more affected by compulsive buying (e.g. Dittmar, 2005a). Schlosser, Black, Repertinger and Freet (1994) report that compulsive buying develops at 18 years of age on average. Consequently the need for research arouses on compulsive buying among young Chinese students for the above mentioned reasons. In the current study, a student sample including students from two Chinese cities – Fuzhou and Chongqing – was investigated.

### Derivation of hypotheses

### Factorial structure

We first tested exploratorily the factorial structure of the translated version of the GCBS for the sample of Chinese students. Thompson and Worthington (2010) emphasized that a higher savings rate compared to the United States or Japan is typical for China. They further demonstrated that the attitude of young Chinese consumers is marked by a pronounced fear of losing control over money spending, especially in the case of using a credit-card. Due to the above described saving characteristics of Chinese consumers, we anticipated that this aspect would play a role in Chinese buying behavior. We tested factorial structure in an exploratory manner. Factorial structure is also a matter of possible measurement invariance. However, prior studies that have used GCBS show a somewhat inconsistent picture according to factorial structure, which is outlined in the discussion.

### Gender

Gender difference is one stable observation in compulsive buying research. Many studies have reported a higher compulsive buying tendency for women (Black, 2001; Dittmar, 2005a, 2005b; O’Guinn & Faber, 1989; Scherhorn et al., 1990). We assumed in this study that Chinese female students would hold a higher compulsive buying tendency than males.

### Location

Furthermore, location may also play a certain role. Usually, distinct consumer culture – along with different available financial resources – interacting with deficiencies of personality is responsible for an increase in compulsive buying. For example, Neuner, Raab and Reisch (2005) found that the percentage of compulsive buyers in the eastern part of Germany increased dramatically after the reunification in 1991. Therefore, different locations along with distinct consumer culture may affect compulsive buying behaviors. Although the locations of our study, Chongqing and Fuzhou, are far distant from each other and fall into different categories of the degree of modernization in ascending manner between third, second, and first tier cities, we assumed that there would not be any significant difference between our two subsamples based on the presupposition that students of the same generation are similarly socialized and share similar experiences with consumption possibilities at the beginning of the 21^st^ century in China.

### Summary of hypotheses

Besides factorial structure, which was tested in an exploratory manner, we tested the following hypotheses:

H1: Female students will show a higher compulsive buying tendency.

H2: Students in Fuzhou and Chongqing will show no significant differences in compulsive buying tendencies.

H3: Female students will show a higher percentage of compulsive buyers.

H4: The percentage of students suffering from compulsive buying in Fuzhou and Chongqing will show no significant difference.

## METHODS

### Participants

We selected two universities, Fuzhou University in Fuzhou (located in Fujian Province) and Southwest University in Chongqing (a municipal city directly ruled by the central government), as the resources of participants in our study. We sampled 384 students from Fuzhou University and 275 from Southwest University, majored in various courses like business administration, public finance and banking, psychology, and architecture. Altogether 659 participants finished the Compulsive Buying Scale, among whom 399 were females and 241 were males (a further 19 did not indicate their gender). Thus, the gender compound in the overall sample was 62.3% female and 37.7% male (not considering the 19 who did not report their gender). According to the subsample from Chongqing, the gender compound was 58.1% female and 41.9% male (3 non-respondents); whereas the compound in Fuzhou was 65.5% female and 34.5% male (16 non-respondents). The reported age of these students ranged from 18 to 25 years, with a mean value of 21.71 (*SD* = 1.83).

### Measures

Compulsive buying tendency in this study was measured by a 16-item self-assessment scale – German Compulsive Buying Scale (GCBS) – compiled by Raab, Neuner, Reisch and Scherhorn (2005), which holds a four-point Likert evaluation standard, ranging from “I don’t agree” (1) to “I totally agree” (4). The GCBS was originally adapted from a Canadian compulsive buying scale developed by Valence, d’Astous and Fortier (1988).

First, we translated the scale into Chinese by using the back-translation method. Further, earlier versions of the items were discussed by a bilingual Chinese team in terms of meaning and wording. In the case of problematic translations and ambiguous meanings, the items were modified stepwise (cf. APPENDIX for English version of GCBS). The original item 9 is, “Advertising or sales letters are interesting for me and often I order something.” We express it in the final version as “Online purchase is interesting for me, and I often shop online” in order to conform more to Chinese conditions. The socio-demographic variables of gender and age are required at the end of the scale. We adopted the cut-off value 45 to identify those who are at high risk to be compulsive buyers, as established in the validation study of the GCBS by Raab et al. (2005). An alternative procedure would be the application of the two-standard deviation rule. This practice suffers, however, from a decisive shortcoming by identifying in each population the identical percentage of compulsive buyers, regardless of the actual prevalence. Raab et al. (2005) have shown that the defined cut-off value 45 is suitable and reliable in identifying those individuals who are at high risk of being compulsive buyers. They report a pre-study that tested successfully if the threshold value could identify a group of clinical compulsive buyers (cf. Raab et al., 2005, p. 51). Similarly, prior supporting results stemmed from a study by Scherhorn et al. (1990).

### Procedure

We selected a convenient sample based on randomly chosen self-study rooms in school buildings. It was declared that the participation was not obligatory and no disadvantage would arise from refusal of participation. Nonetheless, a very small number of 7 students (Fuzhou) and 10 students (Chongqing) refused to participate. In addition, the students invited to participate in this research were told that their data would only be used for the scientific studying purpose. At both universities, the participants filled out the questionnaire in a quiet atmosphere under the supervision of the examiners. Further they were instructed not to talk or even debate during the group-sessions. It was ensured that the participants did not see their neighbors’ responses. The responding time was about 8 minutes. Finally 384 valid data from Fuzhou and 275 from Chongqing were collected.

### Statistical analysis

All of the data were analyzed with SPSS 20.0. First, we conducted an exploratory factor analysis. Second, we reported Cronbach’s *a* and an analysis of reliability, followed by an ANCOVA to test hypotheses 1 and 2. Finally, we applied binary logistic regressions (cf e.g. King, 2008; Tranmer & Elliot, 2008) to test hypotheses 3 and 4, according to the probability of being at high risk for compulsive buying. The predicted probabilities were, as usual, calculated by the following formula: shekar where *e* stands for Euler’s number, *B* for the coefficients (of the constant and factors), and *Xi* for each independent variable. The *B*-coefficients in turn were calculated by the natural logarithm of the odds ratios (ln [odds ratios]). These *B*-coefficients are also named logits (cf. for calculations and further information Cabrera, 1994; Pampel, 2000; Simonoff, 2012, p. 3).

### Ethics

The corresponding researcher’s institutional review boards of all of the involved research institutions approved the design of the survey and declared it in accordance with ethical standards. At the end of data collection all subjects were provided with information about the purpose of the study.

## RESULTS

First, we conducted an exploratory factor analysis (EFA) with varimax-rotation and Kaiser-normalization (Table 1). The extraction was based on the eigenvalue criterion. The Kaiser-Myer-Olkin measure of sampling adequacy was .871, and the Bartlett’s test of sphericity reached significance (<00.001). The EFA resulted in three factors. The first factor (items 1, 2, 3, 4, 5, 8, 13, 14) could be described as combining internal drives (indicating impaired impulse control) and reactive elements (which also involve compensatory aspects). The second factor (items 9,10,11,12) seemed to reflect irrationality in money spending, whereas the third factor clearly reflects post-purchase guilt. The eigenvalues of these three factors were 3.38, 2.19, 1.83, respectively. The total variance explained rate of all three dimensions was 46.25%. The first factor explained 21.09% of the variation, the second 13.70%, and the third 11.46%.

**Table 1.** Factor loading and communality of each item

Item	Factor loading			Communality
	1	2	3	
1	.**505**	.309	–.168	.379
2	.**706**	.167	.129	.543
3	.**708**	.021	.166	.530
4	.**654**	.098	.064	.442
5	.**545**	.161	.097	.332
6	.161	–.052	.**667**	.474
7	.051	.147	.**607**	.392
8	.**512**	.269	.230	.387
9	.035	.**480**	.353	.356
10	.105	.**706**	.190	.546
11	.175	.**782**	.050	.645
12	.311	.**696**	.043	.584
13	.**654**	.095	.143	.457
14	.**672**	.072	.254	.522
15	.153	.134	.**649**	.463
16	.247	.279	.**458**	.348
	Factor correlations			
Factor 1	–			
Factor 2	.45	–		
Factor 3	.41	.38	–	

*Note:* The three factors are named as “impaired impulse control” (factor 1), “reduced rationality in money spending” (factor 2), and “post-purchase guilt” (factor 3), respectively. We conducted varimax rotation with Kaiser-normalization. All 16 items are presented in the APPENDIX.

As shown in Table 1, medium strong, but non-ignorable factor correlations between 0.38 and 0.45 were observed. In this case many authors argue that it is useful to apply oblique instead of orthogonal rotations. Thus, we conducted an additional second factor analysis using oblique rotation. Several types were discussed in the literature (Dien, 2010; Gorsuch, 1970). Here we displayed factorial structures by using direct oblimin and promax rotations (cf. Table 2).

**Table 2.** Factor loading of each item for two oblique rotations

	Factor loading (direct oblimin)			Factor loading (promax)		
Item	1	2	3	1	2	3
1	**.522**	.371	–.098	**.520**	.393	–.046
2	**.736**	.311	.205	**.737**	.346	.261
3	**.717**	.174	.231	**.717**	.211	.281
4	**.664**	.225	.131	**.662**	.258	.181
5	**.573**	.271	.158	**.574**	.297	.203
6	.246	.072	**.673**	.257	.081	**.674**
7	.165	.236	**.618**	.178	.238	**.620**
8	**.580**	.388	.295	**.585**	.412	.340
9	.172	**.523**	.389	.185	**.519**	.408
10	.257	**.733**	.251	.269	**.731**	.287
11	.319	**.800**	.124	.328	**.800**	.169
12	.435	**.741**	.123	.442	**.749**	.174
13	**.674**	.233	.208	**.675**	.266	.257
14	**.704**	.229	.319	**.706**	.264	.367
15	.269	.249	**.669**	.282	.256	**.677**
16	.358	.381	**.498**	.369	.391	**.522**
	Factor correlations			Factor correlations		
Factor 1	–			Factor 1	–	
Factor 2	.38	–		Factor 2	.44	–
Factor 3	.25	.23	–	Factor 3	.34	.29 –

*Note:* We conducted direct oblimin and promax rotations with Kaiser-normalization. All 16 items are presented in the APPENDIX.

The three-factorial structure remains the same for both. The assumption of oblique relations of the axis of the three dimensions may be an approach to integrate observations of one-dimensional (e.g. Raab et al., 2005) and multi-dimensional factor structures (e.g. Scherhorn et al., 1990). It would imply the existence of the three distinguishable factors, which show – however modest – correlations to each other. This, nonetheless, has to be investigated in future research.

According to the reliability of the full-16-item version, the Cronbach’s a in this study was .83, of which the three dimensions’ were .81, .67 and .59, respectively. This indicated good reliability for the full-16-item version, as well as for the first identified factor. Both the two other dimensions, however, suffered from limited reliability. We further conducted an analysis of the 16 items including facility index, selectivity coefficient (by using *r*_s_), and Lienert’s selection index (cf. Table 3, which also shows the descriptive statistics of the items). The facility index (also named difficulty index) implies (e.g. Mitra, Nagaraja, Ponnudurai & Judson, 2009) the measurement of how many persons respond in favor of an item. In general, it is recommended that the percentage should be between 20% and 80% (Zöfel, 2003, p. 235). Those below and above are not informative because either almost all reject the item (20% or below), or almost all are in favor of the item (80% or above). In the case of the multi-stage scale, the following formula is used for calculating the facility index: Shekar *P_j_* = (*M_j_ – X_min_*)/(*X_max_ – X_min_*) * 100, whereas Shekar *X_min_* and *X_max_* stand for the lowest and highest possible values of the scale (1 and 4 in the case of GCBS). The Lienert’s selection index (Lienert & Raatz, 1994) is based on the quotient of the discriminatory index and the twofold standard deviation of each item, whereas the discriminatory index is calculated by using the spearman-rank-correlation coefficient between the corresponding item and the overall-index (by omitting the item at stake for the calculation of the particular overall-index).

**Table 3.** Analysis of items of the Chinese translation of the German Compulsive Buying Scale (GCBS)

Item	Mean	*SD*	Item total correlation *(r_s_)*	Facility index	Lienert’s selection index
1	1.712	0.826	0.356	23.67%	0.215
2	2.181	0.932	0.561	39.33%	0.301
3	2.325	1.008	0.513	44.00%	0.254
4	2.472	0.997	0.456	49.00%	0.229
5	2.376	1.001	0.427	46.00%	0.213
6	2.519	1.030	0.294	50.76%	0.143
7	2.329	0.961	0.285	44.33%	0.148
8	1.967	0.937	0.529	32.33%	0.282
9	1.826	0.920	0.347	27.67%	0.189
10	1.622	0.757	0.434	20.67%	0.287
11	1.384	0.604	0.441	12.67%	0.365
12	1.800	0.832	0.488	26.67%	0.293
13	2.052	0.963	0.500	35.00%	0.260
14	2.080	0.907	0.553	36.00%	0.305
15	2.205	0.981	0.357	40.00%	0.182
16	1.686	0.838	0.474	23.00%	0.283

*Note:* All 16 items are presented in APPENDIX.

**Table 4.** Analysis of covariance for gender and location, with age as covariate

Source	*df*	*MS*	*F*	*p*	*η*_p_^2^
Age (covariate)	1	22.44	0.40	.529	.001
Gender (G)	1	2014.46	35.61	.000	.055
Location (L)	1	337.98	5.98	.015	.010
G × L	1	65.61	1.16	.282	.002
Error	608	56.57			

The facility index for item 11 was too low (these values should not exceed 80% or fall below 20%). Further, the selection indexes for items 6 and 7 showed low values. These items should be scrutinized, and modifications should be considered in future research. All other items showed acceptable results.

To test Hypotheses 1 and 2, we conducted an ANCOVA including gender and location of data collection (Chongqing vs. Fuzhou) as factors, and age as a covariate. The sum of scores on the overall 16 items as the compulsive tendency index was the dependent measurement of this ANCOVA. The conducted ANCOVA revealed a significant main effect of gender, shekar *F*(1, 608) = 35.61, *p* < .001; *η*^2^_p_ = .06. Female students showed a higher compulsive buying overall index than men, shekar *M*_female_ = 34.00; *SD* = 7.64 vs. *M*_male_ = 30.29; *SD* = 7.38. And the main effect of location reached significance too, shekar *F*(1, 608) = 5.98, *p* = .015; *η*^2^_p_= .01 (cf. Table 4). A stronger tendency of compulsive buying was observed among the students in Chongqing shekar (*M* =33.21; *SD* = 7.89), compared to the students in Fuzhou shekar (*M* = 32.14; *SD* = 7.62). Thus, Hypothesis 1 according to gender was confirmed. Location, contrary to Hypothesis 2, however, showed a significant influence on compulsive buying tendency, which needs further clarification. Furthermore, the interaction of gender by location did not reach significance, shekar *F*(1, 608) = 1.16, *p* = .282; *η*^2^_p_ = .00, and the covariate of *age* did not reach significance either (*p* = .529).

To test the hypothesis about the percentage of compulsive buyers (hypotheses 3 and 4), we conducted a binary logistic regression, using the simple-contrast-method. The probability to be a compulsive buyer was used as a dependent measurement. We adopted the established cut-off value by Raab et al. (2005). The ones who score at 45 or more were classified as compulsive buyers. We included gender (“male” was defined as reference category) and location (“Fuzhou” was defined as reference category). As predicted, a significant effect of gender was observed, shekar *B* = 0.86; OR = 2.35, 95% CI [1.10, 5.05], *p* = .028. The probability for females was higher compared to males. The interaction of gender by location did not show significance, shekar *B* = –0.55; OR = 0.58, 95% CI [0.13, 2.67], *p* = .485. Contrary to the results for the overall index in the ANCOVA, but in line with our assumption, no significant effects were observed in location for the probability of being a compulsive buyer, shekar *B* = 0.07; OR = 1.07, 95% CI [0.50, 2.31], *p* = .854 (cf. Table 5).

**Table 5.** Binary logistical regression “gender and location” predicting percentage of compulsive buying

Variable	*B*	*SE*	*OR*	95% CI	Wald statistic	*P*
Gender (1)	0.86	0.39	2.35	[1.10, 5.05]	4.82	.028
Location (1)	0.07	0.39	1.07	[0.50, 2.31]	0.34	.854ti
G(1) × L (1)	–0.55	0.78	0.58	[0.13, 2.67]	0.49	.485

*Note:* For gender “male” and for location “Fuzhou” was defined as reference category.

The calculated percentage of those being identified as potential compulsive buyers was 6.7% in the overall sample. The percentages of Fuzhou and Chongqing were 7.0% and 6.2%, respectively, which were calculated on the basis of the predicted probabilities of the separate binary logistic regression, including only the variable location; shekar *B* = –0.14; OR = 0.87; 95% CI [0.47, 1.63], *p* = .667.

## DISCUSSION

The main purpose of this study was to give a first insight to Chinese young consumers’ compulsive buying behavior and provide some preliminary information about the applicability of GCBS in China.

Previous studies have shown that the construction of compulsive buying did not remain necessarily consistent in different countries. The original GCBS declared one dimension in investigating a German sample (Raab et al., 2005). However, several studies using the GCBS or the prior Canadian version (which the GCBS is based on and shares most identical or quite similar items with) reported deviations from the one-dimensional pattern as outlined in more detail later.

To examine how the factorial structure of GCBS could be described, an EFA was carried out. The result showed a meaningful factorial structure with three interpretable factors for Chinese young consumers. The main factor (items 1, 2, 3, 4, 5, 8, 13, 14) reflects external and internal impulse, thus capturing buying tendency as well as reactive or compensatory aspects which could be labeled as “impaired impulse control”. The second factor (items 9, 10, 11, 12) seems to reveal irrationality in money spending, therefore, it is labeled as “reduced rationality in money spending”, although item 9 did not entirely fit in our categorization. The dimension may reflect violations of long-term thrifty orientation in Chinese culture and existing norms of rational and moderate behavior in money spending, as well as financial obligations towards future family. Previous studies have shown that China has a typically higher savings rate (Thompson & Worthington, 2010), and that sustainable orientation gives Chinese consumers a reluctant spending behavior (Li, Jiang et al., 2009). Future research could be done to clarify if this dimension indeed reflects a perceived deviation from the commonly strong awareness of obligation and savings for Chinese consumers. Then “post-purchase guilt”, which was well observed in the previous studies (Cole & Sherrell, 1995; Scherhorn et al., 1990), appeared as the third factor (items 6, 7, 15, 16). Thus, besides the first and third factor, which are similar to prior observations (Scherhorn et al., 1990), we have observed one specific factor for Chinese students – reduced rationality in money spending. In this study, Chinese consumers have obtained a different factorial structure with the one-dimensionality reported by Raab et al. (2005) for Germans, which indicated that we could not replicate the same one-dimensional pattern in Chinese consumers, as it would be assumed for scale equivalence.

Several studies using the GCBS as well as the Canadian version reported multi-dimensional factor structures of compulsive buying. A study by Cole and Sherrell (1995) analyzed and compared the dimensionality of Compulsive Buying Measurement Scale (Valence et al., 1988) and the Compulsive Buying Scale (Faber & O’Guinn, 1992). According to the Canadian Compulsive Buying Measurement Scale, three dimensions were identified. The authors named the dimensions as (a) tendency to spend; (b) reactive aspect; and (c) post-purchase guilt. The study shows that for the precursor Canadian version, not every study conforms to the observation of one-dimensionality of the scale, as reported by Valence et al. (1988). Further, Scherhorn et al. (1990) observed a multi-dimensional pattern by conducting a factor analysis for their German version. Their results and interpretation seem valuable to be outlined here in detail. These authors investigated two samples, an inconspicuous group and an extreme group consisting of individuals who reported themselves at a high risk for being a compulsive buyer. For the “normal” group they identified one dominant main factor by using a scree plot and they even identified this factor as the single factor. This procedure is, however, described as highly subjective. By conducting an exploratory factor analysis, the authors identified four factors explaining 59.5% of the variance for the “normal” control group; and for the extreme group they observed three factors (however, the authors declared that even the forth factor should be considered since its eigenvalue is over 1, but they only reported the former three factors’ explaining rate was 65.9%.) (cf. Scherhorn et al., 1990). As a main difference, these authors reported for the extreme group “[…] an internal and an external component, represented by factors 1 and 2, respectively […]” (Scherhorn et al., 1990).

Taken these results together one could see that the reported factorial pattern of compulsive buying is not yet clarified in an unambiguous way and it varies with different samples. In the current study, Chinese consumers showed a three-factorial structure, which is distinct from that of the westerners in compulsive buying. Future research is needed to clarify whether this inconsistent observation indeed reflects a different compulsive buying pattern for Chinese students or it is caused by measurement biases (cf. van de Vijver & Leung, 1997).

The current study shows some severe limitations, but also delivers some preliminary information about the compulsive buying behavior of Chinese students. Our study shows a possible factorial structure with three meaningful interpretable dimensions for Chinese consumers; which, however, has to be further tested in follow-up studies for a possible invariance of measurement. In addition, validation studies for the GCBS in China are needed, especially cross-validations with constructs like low self-esteem, materialistic values and other related variables. It would be quite useful because the issue of compulsive buying in China’s booming economy is of growing interest (cf. Chang, Lu, Su, Lin & Chang, 2011; Guo & Cai, 2011; Li, Jiang et al., 2009; Li, Yang & Wang, 2009; Podoshen, Li & Zhang, 2011; Unger & Raab, in press), although validated scales for China are still missing.

Furthermore, our results have indicated that just like in Western settings (e.g. Dittmar, 2005a) there is a higher prevalence of female consumers in China. Also, the gender differences in compulsive buying may be highly related to females’ role in Chinese society, most of them are responsible for getting the daily use products for the family, and they have relatively more time to spend in supermarkets and shopping malls. Although more and more modern females have their own careers nowadays, they could still be influenced by their mothers, thus acquiring similar buying patterns according to the social learning theory (Hirschman, 1992). On the other hand, the financial obligations like responsibility for the family and necessity to save money for marriage (cf. also Unger & Raab, in press) may work as triggers that inhibit the overconsumption tendency, especially for young Chinese male consumers.

The results about the observed unexpected geographic difference indicated that local characteristics should be considered. The significant difference may result from different economic situations in which the students are immersed, different pronounced consumer and materialistic orientations dependent on the degree of modernization, and size of the city where the university is located. However, it has to be further tested for a clearer view of the geographic point.

To give a summary, we can state that the current study contributes to the *preliminary* identification of the three described dimensions for Chinese compulsive buying behavior, two of which (except the observed factor “reduced rationality”) highly resemble the dimensions identified by Scherhorn et al. (1990) and Cole and Sherrell (1995). However, the factorial structure was contradictory to those studies showing one-dimensionality like Valence et al. (1988) for the Canadian case, and Raab et al. (2005) for the German adaption. A possible resolution might be the assumption of an oblique instead of orthogonal relationship between the factors. In addition, prevalence rates of compulsive buying were reported and some valuable observations about gender and location were considered. Follow-up studies and validating studies could further increase the understanding of compulsive buying in China.

## Figures and Tables

**Table:** 

1.	When I have money, I cannot help but spend part or all of it.
	I don’t agree 1 2 3 4 I totally agree
2.	As soon as I enter a shopping center or mall, I have an irresistible urge to go to a shop and buy something.
	I don’t agree 1 2 3 4 I totally agree
3.	I often have an unexplainable urge, a sudden and spon taneous desire, to go and buy something.
	I don’t agree 1 2 3 4 I totally agree
4.	Sometimes I see a product and feel a strong irresistible impulse to buy it.
	I don’t agree 1 2 3 4 I totally agree
5.	Often I have the feeling to own a certain item by any means.
	I don’t agree 1 2 3 4 I totally agree
6.	After purchase I often ask myself if this purchase was really necessary.
	I don’t agree 1 2 3 4 I totally agree
7.	I often buy something, simply because it is cheap.
	I don’t agree 1 2 3 4 I totally agree
8.	There are times when I go shopping just for fun.
	I don’t agree 1 2 3 4 I totally agree
9.	Online purchase is interesting for me and I often shop online.*
	I don’t agree 1 2 3 4 I totally agree
10.	I often have bought something that I did not use at all afterwards.
	I don’t agree 1 2 3 4 I totally agree
11.	I have often bought a product that I did not need, even when I knew that I had very little money left.
	I don’t agree 1 2 3 4 I totally agree
12.	I spend money lavishly.
	I don’t agree 1 2 3 4 I totally agree
13.	For me, shopping is a way of escaping the stress of my daily life and relaxing.
	I don’t agree 1 2 3 4 I totally agree
14.	I sometimes feel that something inside pushed me to go shopping.
	I don’t agree 1 2 3 4 I totally agree
15.	At times, I’ve felt somewhat guilty after buying a prod uct, because it seemed unreasonable.
	I don’t agree 1 2 3 4 I totally agree
16.	There are some things I buy that I do not show to any body because I’m afraid people will think I wasted my money.
	I don’t agree 1 2 3 4 I totally agree

* Modified item; the original item of GCBS refers to sales letters, which are less in use in China.

## APPENDIX

### THE GERMAN COMPULSIVE BUYING SCALE (GCBS)

This questionnaire is about your purchase behavior. Please read carefully each question. There are no wrong answers. Please decide for each statement to which extent you agree or disagree for you personally, by circling the appropriate number.
